# Supplementary material for: Chromothripsis during telomere crisis is independent of NHEJ, and consistent with a replicative origin
Source: Genome Res. 2019 May;29(5):737–49. doi: 10.1101/gr.240705.118 (PMC6499312; doi:10.1101/gr.240705.118)
Supplement: Supplemental Material [file supp_gr.240705.118_Supplemental_file_1.zip › contigs/annotated_contigs/DB79/contig.2.DB79_length_604_mean_cov_8.19536423841.docx]

**DB79_length_604_mean_cov_8.19536423841**

```
GGACCAAAACTCCCATCTTCCTCTGGAAATCTCTCCTCCCAGAGCCCCTACGCTGGCCTGGGGGCGCTGCTGCCCCTCACGTCCACAGC
 >chr9:138290736-138291098 - E=4e-203
TGGTGGCCCGGCTGCTCCCTCTGTATGCCTCATCTCCTCTTTCCACCCTCGTTGCTCAATGTTGCCCCCAACTCTAGCCTGGACCCTGA

AATGGTCCCCTTCCGGCTCCCTCAGGCAGTAGGTGCTCTCCCTAACTCCTCACTCTGACCACATCCGTCTCCACCAGAGCCCACAGCTC

CCCGGGCCCAGGACCCCCAGTGTGGTCCCCGCTCACCCGGCCTGGATGGCTTCTGCCTACCCAGAGCTGCCTAGTGTGGCTTCCCTACC

CGGCCC|A|TGTAATTTTCTTTTCATGTAGTCTCTTTGTTTGGCTTTGCTATCAGGGTAATGCTGACCTCACAAAATGAGTTTGAAAGT
        >chr9:123566667-123566908 + E=3e-127
ATTCTCTCTTCTGTTTTGAGGGGAGAGTTTGAGAAGGATTGGTATTAATTCTTCTTTAAGTGTTTGGTAGATGGCTTCAGCAGTTGGTG

GGAGATTTTTTATTGCTGATTCAATCTCTTTACTCTGTGCTGGTCGGTTCAGATTTTCCATTTCCTCATGAT
```
